# Blackseed Oil Supplemented Caseinate–Carboxymethyl Chitosan Film Membrane for Improving Shelf Life of Grape Tomato

**DOI:** 10.3390/ma18112653

**Published:** 2025-06-05

**Authors:** Amal M. A. Mohamed, Hosahalli S. Ramaswamy

**Affiliations:** Department of Food Science and Agricultural Chemistry, Macdonald Campus of McGill University, 21111 Lakeshore Road, Ste Anne de Bellevue, QC H9X 3V9, Canada; amal.mohamed@mail.mcgill.ca

**Keywords:** edible film, membrane, composites, coating, tomato, shelf life, quality

## Abstract

Blackseed oil supplemented with caseinate (CA)–carboxymethyl chitosan (CMCH) composite membranes were evaluated for their functional properties and as edible coating for extending the shelf life of grape tomatoes. Composite films were prepared from equal parts of (CaCa or NaCa) and (CMCH) with or without supplemented 3% blackseed oil (BO) and evaluated for their functional properties. Subsequently, the edible membrane coating was evaluated to extend the shelf life of grape tomatoes (*Solanum lycopersicum* L.). The water vapor permeability (WVP) of the films was the lowest for the calcium caseinate–carboxymethyl chitosan–blackseed oil (CaCa-CMCH-BO) film (3.01 g kPa^−1^ h^−1^ m^−2^). Adding blackseed oil to the edible film matrix also led to a significant increase in its mechanical properties, resulting in tensile strength values of 12.5 MPa and 10.2 MPa and elongation at break values of 90.5% and 100% for NaCa-CMCH-BO and CaCa-CMCH-BO, respectively. The composite films also exhibited good compatibility through hydrogen bonding and hydrophobic interactions, as confirmed by FTIR spectroscopy. The particle size and zeta potential of CaCa-CMCM-BO were 117 nm and −40.73 mV, respectively, while for NaCa-CMCH-BO, they were 294.70 nm and −25.10 mV, respectively. The incorporation of BO into the films resulted in greater antioxidant activity. When applied as an edible film membrane on grape tomatoes, the coating effectively delayed the deterioration of tomatoes by reducing weight loss, microbial spoilage, and oxidative degradation. Compared to the control, the coated fruits had delayed ripening, with a shelf life of up to 30 days, and reduced microbial growth over the entire storage period.

## 1. Introduction

Offering a sustainable alternative to plastic packaging due to its biodegradability and environmentally friendly characteristics, natural biopolymers are a promising solution for creating edible food packaging films [1]. Edible films have been developed using biomolecules like polysaccharides, proteins, and lipids, either individually or in combination, which define their functional properties [2]. Proteins and polysaccharides are particularly notable for their transformation into environmentally friendly packaging materials. These biopolymers are effective in the formation of films and coatings, which exhibit desirable properties when applied to food products. Further, edible biopolymer films have unique properties, making them a desirable matrix for incorporating different additives that improve their ability to serve as barriers against moisture, gases, and aromas. The incorporation of natural antimicrobials and antioxidants into these films can provide additional protective effects, ensuring safety and quality. Edible films and coatings can be amended with proteinaceous materials, offering a further option for generating renewable, edible, and sustainable alternatives that can help reduce secondary packaging wastes by being consumed with the product.

Casein and other milk products can be used to produce films and coatings. Offering good strength and low flexibility, casein films have been shown to be effective oxygen barriers, protecting foods from oxidation. However, such films are highly sensitive to moisture. Casein can be utilized in a variety of commercial applications by modifying the films to suit different environmental conditions. Biodegradable film materials have also been derived from polysaccharides, including carboxymethyl chitosan (CMCH), a modified form of chitosan that dissolves easily in water. Given its excellent ability to form a film, its biodegradability, non-toxicity, safety, antibacterial activity, and biocompatibility, CMCH has been extensively studied as an edible film material for coating food products like fruits and vegetables. However, offering a weak barrier, CMCH films are very brittle, absorb water easily, and their mechanical properties limit their sole use in food packaging [3]. To make CMCH films more useful in the food industry, it is necessary to improve their physical properties (e.g., flexibility, barrier, and mechanical strength).

A widely used approach to enhance and improve a film/coating’s functional properties is to incorporate additives or blend it with other polymers like blending proteins and polysaccharides. This approach can lead to the development of stronger, more flexible, and more effective materials that meet desired final properties and are suitable for the specified application. For example, Rachtanapun et al. [4] found that blending gelatin with CMCH enhanced the resultant film’s mechanical properties. Using microalgae protein-CMCH complexation, Wang et al. [5] improved the protein’s emulsification capacity and enhanced its viscosity. Moreover, Li et al. [6] found that incorporating soy protein with CMCH improved the resultant film’s tensile strength and water resistance, making this combination a good option for packaging applications.

Having recently gained significant attention, another method to improve the multifunctional properties of blended edible films is by incorporating essential oils into the film matrix. Essential oils with antimicrobial properties have attracted significant interest in the food industry, especially in the food packaging sector, for their ability to extend food shelf life and enhance food safety. Essential oils, including cinnamon (*Cinnamomum verum*) [7], rosemary (*Salvia rosmarinus*) and aloe vera [8], and *Ziziphora clinopodioides* Lam. [9], have been found to enhance antimicrobial activity and physicochemical properties of edible films.

*Nigella sativa* L., from which blackseed oil (BO) is produced, is an annual flowering plant of the *Ranunculaceae* family. It grows in Eastern Europe, the Middle East, and Western Asia. The plant produces small blackseeds that are flat, trigonous, and angular in appearance. The blackseeds and their oil have been recommended as therapy for rheumatoid arthritis, asthma, inflammatory diseases, diabetes, and digestive diseases. *N. sativa* seeds contain unsaturated fatty acids (26–38%), proteins, alkaloids, saponins (melanin), and essential oils (0.4–2.5%) [10]. The essential oil components of BO have been shown to have anti-cancer, antioxidant, gastroprotective, hepatoprotective, analgesic, anti-inflammatory, antihypertensive, antidiabetic, and antimicrobial impacts [11]. The antimicrobial and antioxidant potential of BO makes it an excellent candidate for use as an active agent in food packaging.

The edible berries of tomato (*Solanum lycopersicum* L.) have become an essential ingredient in the cuisines of many countries worldwide and can be consumed either raw or cooked. Tomato is the second most commercially significant vegetable crop globally, following potato (*Solanum tuberosum* L.), with an annual production of approximately 163 Tg [12]. Tomatoes are a rich source of essential vitamins (i.e., A, B, C, and E) and numerous bioactive compounds, including carotenoids (lycopene and β-carotene), chlorophyll, organic acids, flavonoids, and phenols, which contribute to health benefits like reducing the risk of cardiovascular diseases [12]. Tomatoes have a relatively short postharvest shelf life due to factors such as transpiration, diseases, ripening, and senescence. These issues pose significant challenges for their marketing and result in postharvest losses of up to 50%. To mitigate these losses and extend shelf life, several innovative preservation techniques have been used. Among these methods, edible coatings have emerged as a promising solution to maintain tomato quality and reduce food waste while meeting growing consumer demand for fresh produce. The most important feature of an edible coating is that it can be consumed along with the fruit or vegetable that was coated, and the original nutrients are always retained [13].

The main objective of the present work was to explore the effect of incorporating BO into caseinate–carboxymethyl chitosan edible film formulations to enhance their functional properties, including barrier and mechanical characteristics, as well as antioxidant activity. The study was designed to investigate the effects of bioactive compounds in blackseed oil, when combined with biopolymers, on extending the shelf life of grape tomatoes, thereby demonstrating their potential in food preservation and packaging.

## 2. Materials and Methods

Carboxymethyl chitosan and caseinates were obtained from Nutra Key Industries, Inc. (Qingdao, China) and CALDIC Canada Inc. (Mississauga, ON, Canada), respectively. Both grape tomatoes and blackseed oil were purchased from local stores in Montreal, Canada. Glycerol was obtained from Bulk Apothecary (Aurora, OH, USA).

### 2.1. Preparation and Characterization of Edible Membrane/Film/Coating

#### 2.1.1. Preparation of Edible Film

The two solutions were first prepared to create edible membranes/film/coating material. A 50 mL of 2% carboxymethyl chitosan (CMCH) was first mixed with 50 mL of 8% sodium caseinate (NaCa) for the first solution and a 50 mL of 2% carboxymethyl chitosan with 50 mL of 8% calcium caseinate (CaCa) for the second one. Following these preparations, 5% *w*/*w* of glycerol and 0.1% *w*/*w* Tween 20 were added, and the solution was mixed for 10 min. Subsequently, 3% blackseed oil (BO) was incorporated and homogenized at 13,000 rpm for 15 min. This ratio was selected based on prior studies demonstrating that a (1:1) balance of the two polymers improved the mechanical properties, flexibility, and barrier characteristics. Additionally, 3% of blackseed oil was chosen based on preliminary trials.

The film-forming solutions were then cast by pouring 14 mL of each film solution into Petri dishes and drying at 45 °C for 8 h. The films, all bearing the same quantity of carboxymethyl chitosan, combined with either Na or Ca caseinate with and without blackseed oil, were labeled as follows:

NaCa-CMCH-BO: Sodium caseinate–carboxymethyl chitosan with blackseed oil

NaCa-CMCH: Sodium caseinate–carboxymethyl chitosan without blackseed oil

CaCa-CMCH-BO: Calcium caseinate–carboxymethyl chitosan with blackseed oil

CaCa-CMCH: Calcium caseinate–carboxymethyl chitosan without blackseed oil

#### 2.1.2. Preparation of Sample Coating

A total of 500 mL of each coating solution was prepared to coat the grape tomatoes. Overall, 6 kg of grape tomatoes were used. The coating procedure was executed using the dipping method. The tomatoes were first washed with water to eliminate any dust or oil. This was followed by their immersion in a 0.1% sodium hypochlorite solution for 5 min and air drying at room temperature. The tomatoes were divided into three groups: one remained uncoated (control), one was coated with NaCa 50-3%BO and another with CaCa 50-3%BO. The tomatoes were left in the coating solution for 5 min and then drained and dried. All samples were then kept refrigerated at 4 °C. Fruit testing was performed on days 0, 7, 14, 21, 28, and 45 days.

## 3. Characterization of Films

### 3.1. Physicochemical Properties

#### 3.1.1. Particle Size and Zeta Potential Measurement

The particle size and zeta potential of the film samples were diluted with water at a ratio of 1:10; then, they were measured by dynamic light scattering (DLS) (Nano Brook, OMNI instrument, South Plainfield, NJ, USA) at 25 °C and applied with a voltage of 100 V. Each sample was analyzed on at least two occasions and the average was taken to determine the structure of film-forming emulsions.

#### 3.1.2. Thickness

The thickness of the film samples was measured manually at ten different random points of the film sample using a digital micrometer (Mitutoyo Manufacturing, Tokyo, Japan) with a sensitivity of 0.1 μm. The test was performed in triplicate, and the average values were calculated for each film.

#### 3.1.3. Moisture Content

The moisture content was determined through a modified version of the method of [14]. Film samples were cut into 2.5 cm × 2.5 cm squares and then weighed. They were then dried at 105 °C for 24 h. The dried samples were weighed, and the moisture content (MC) of the edible film was calculated as:

$$MC(\%)=100\cdot \frac{FW-DW}{FW}$$

where *DW* is the dried weight of the sample, and *FW* is the initial fresh weight of the sample.

#### 3.1.4. Water Solubility

The water solubility (WS) of the film samples was determined as the percentage of dissolved film after immersion of dried films in distilled water for 24 h. The edible films were cut into a triangular shape (1 cm × 4 cm) and then dried at 105 °C for 24 h to attain the initial dry weight (*DW*_init_). The dried samples were immersed in distilled water (50 mL) and placed on a shaker for 24 h. The remaining portion of the samples was dried once again to obtain the final dry weight (*DW*_final_). The WS (%) was then calculated [14,15].

Water solubility (WS) % = [(DW_init_ − DW_final_)/DW_init_] × 100

#### 3.1.5. Water Vapor Permeability (WVP)

The water vapor permeability (WVP) was determined using the gravimetric method [16]. Anhydrous calcium chloride (5 g) was placed in a cup (3 cm × 5 cm × 7 cm) to maintain a 0% relative humidity. The cups were covered with circular film samples. The cups were placed in a desiccator containing a saturated sodium chloride solution (75% RH). The tests were performed at room temperature. The WVP (g m^−1^ s^−1^ Pa^−1^) of film samples was measured from the weight gain of the cups using the following equation:

$$WVP=\frac{WVTR\cdot L}{∆P}$$

where *WVTR* is the water vapor transmission rate (g m^−2^ h^−1^), calculated from the slope of the regression curve divided by the area (m^2^), *L* is the film’s thickness (m), and ∆*P* is the water vapor pressure differential between the two sides of the film (Pa).

#### 3.1.6. Color and Opacity Index

Color measurements were made using a Minolta Chroma Meter (Minolta Corp., Ramsey, NJ, USA) (illuminant: D65, standard daylight, observer angle 10°, and color space: CIE L*a*b*). The reading was taken at 10 random points on the film surface. L*, a*, b* parameters were used to calculate the total color difference (∆E), where L* is the brightness, and a* and b* are trichromatic coordinates. The reference standard was a white plate with color parameters L* = 96.58, a* = −0.21, b* = 2.11. The ∆E and yellow index (YI) between the film and standard plate were calculated as [17].

$$∆E=\sqrt{{{(L}_{std}^{*}-L_{film}^{*})}^{2}+{{(a}_{std}^{*}-a_{film}^{*})}^{2}+{{(b}_{std}^{*}-b_{film}^{*})}^{2}}$$

$$YI=\frac{142.86\cdot b_{film}^{*}}{L_{std}^{*}}$$

The opacity index of the film samples was measured using an UV/VIS spectrophotometer (VWR, Radnor, PA, USA Model UV-3100 PC). The edible films were cut into pieces (1 cm × 4 cm) and placed in the cuvette, and the reading was taken at 600 nm. The opacity was calculated as

$$Opacity=\frac{A_{600}}{X}$$

where $A_{600}$ is the absorbance of the sample at 600 nm and *X* is the thickness of the film in mm.

#### 3.1.7. Mechanical Properties

The mechanical properties, including tensile strength (TS) and elongation at break (EAB), of the film samples were evaluated according to ASTM standard method 638-10, with slight modifications, utilizing an Instron Universal Testing Machine (Model 4500, Instron Corporation, Canton, MA, USA) [18]. Film pieces measuring 30 mm × 10 mm were prepared and inserted between grips set initially at a distance of 50 mm, with a crosshead speed of 30 mm s^−1^. Testing was performed at room temperature at a relative humidity of 50% ± 5%. Tensile strength (MPa) was calculated using the maximum load and final extension at break, while elongation at break (EAB) was expressed as a percentage. For accuracy and consistency, five replicates of each film were tested.

#### 3.1.8. Antioxidant Activities

##### DPPH Free Radical Scavenging Activity Assay

DPPH free radical scavenging activity of edible films was determined as described by [19] with some modifications. The film sample (5 g) was immersed in a solution of 1:9 distilled water—ethanol—and sonicated for 10 min. The solutions were centrifuged for 15 min at 6000 rpm. The supernatant was collected and mixed with distilled water and an ethanol solution of DPPH. The samples were incubated in the dark for 30 min. For the control sample, distilled water was used instead of a film sample. After incubation, an absorbance reading was taken at 517 nm using a spectrophotometer (UV-3100). The DPPH activity was calculated as

$$DPPH(\%)=100\cdot [1-(A_{517}^{S}-A_{517}^{C})]$$

where $A_{517}^{S}\;and\;A_{517}^{C}$ are, respectively, the absorbance reading of the sample and control at 517 nm.

##### ABTS Radical Scavenging Activity Assay

The radical scavenging activity of the edible film was measured using the ABTS assay [20]. Briefly, a film solution sample of about 30 μL was added to a centrifuge tube containing 3 mL of diluted ABTS solution (A_734 nm_ = 0.7 ± 0.02) and mixed. After 10 min, the absorbance was taken at 734 nm. Water was used instead of film for the blank sample. The ABTS radical scavenging activity (%) was determined as

$$ABTS=100\cdot \frac{A_{734}^{blank}-A_{734}^{sample}}{A_{734}^{blank}}$$

where $A_{734}^{blank}$ is the absorbance of the blank at 734 nm, and $A_{734}^{sample}$ is the absorbance at 734 nm of the sample 10 min after the addition of the ABTS solution.

#### 3.1.9. Structural Properties

##### Fourier Transform Infrared Spectroscopy (FT-IR)

FT-IR of the film samples was carried out using an FTIR spectrometer (Agilent 5500a; Northern ANI Solutions, Suwanee, GA, USA). The measurements were recorded between 400 and 4000 cm^−1^.

##### Scanning Electron Microscopy (SEM)

The morphology of surfaces and cross sections of the films were examined using scanning electron microscopy (SEM) (TM3000, Hitachi Tabletop Microscope, Tokyo, Japan). The film samples were mounted on aluminum stubs using double-sided carbon adhesive tape and photographed at room temperature at a magnification of 600×. The films were observed at an accelerating voltage of 5 kV.

## 4. Physicochemical Properties

### 4.1. pH, Brix, Titratable Acidity, and Weight Loss

The pH of grape tomatoes was measured at 20 °C using a digital pH meter (Brinkman Co., Mississauga, ON, Canada). Tomato samples (50 g) were blended in 150 mL distilled water following AOAC method 981.12 [21]. Total soluble solids were measured using a digital refractometer (ATAGO, Model. PAL-α , Prolabscientific, 1974 Laval, QC, Canada) and expressed on the Brix scale 0–85%. Titratable acidity (TA) was measured using the AOAC method 942.15 [22]. Results were expressed as g citric acid per 100 mL of sample.

Weight loss (WL) was evaluated as described by [23] by weighting coated and uncoated samples with a precision balance from the first to the last day of storage. The percentage weight loss (%) was calculated as

$$WL\;(\%)=100\cdot \frac{{FW}_{t=0}-{FW}_{t=x}}{{FW}_{t=0}}$$

where ${FW}_{t=0}$ is the initial fresh weight of the tomato sample (*t* = 0), and ${FW}_{t=x}$ is the fresh weight of the tomato sample at sampling day *x.*

### 4.2. Color Measurement

Color changes were monitored with a tristimulus Minolta Chroma Meter (Minolta Corp., Ramsey, NJ, USA) calibrated with standard black and white plates. Readings of L* (lightness), a* (Green, red chromaticity), and b* (yellow, blue chromaticity) from ten samples per treatment (coated and uncoated samples) were recorded at room temperature.

### 4.3. Determination of Total Phenolic and Ascorbic Acid

The total phenolic content of uncoated and coated samples was determined using a spectrophotometric method [24]. About 5 g of sample was blended in a homogenizer, and then 20 mL of menthol was added. The sample was placed in a hot water bath for 24 h. In a tube containing 3 mL of deionized water, 50 μL of the sample extract and 250 μL of a Folin–Ciocalteu reagent were added. The mixture was incubated for 5 min, and then 750 μL of Na_2_CO_3_ (20%) was added. The solution was brought to 5 mL with deionized water. The phenols were measured at 760 nm after a 30 min incubation in the dark. The results were expressed as mg GAE g^−1^.

The determination of ascorbic acid (AA_s_) was performed based on the titration method (DCPIP) employed by [24,25], with some modifications. Each 10 g sample of tomato was blended in 3% metaphosphoric acid to make a final volume of 100 mL. Five milliliters of the sample extract were titrated with 2,6-dichlorophenolindophenol dye to a pink endpoint color. A standard curve of AA_s_ was used at different concentrations and expressed as mg of ascorbic acid per 100 g of fresh weight.

### 4.4. Texture Evaluation

Measuring food texture before and after treatment is a critical step in food processing and quality control. It involves assessing how the texture of a food product has been modified due to various treatments. The relevant parameters are hardness (N), defined as maximum force at the first compression peak, and chewiness (N·mm), defined as the total amount of work necessary to chew a sample to a state ready for swallowing. Coated and uncoated samples stored at 4 °C were analyzed using a TA.XT. *Plus* Texture Analyser (Texture Technologies Corp., Scarsdale, NY, USA) using a texture profile analysis (TPA) device equipped with a 25 mm diameter cylindrical probe, a 50 kg load cell, operating in compression mode. Textural analysis methods followed those of [26], with some modifications. The test profile had a pre- and post-test speed of 2.0 mm s^−1^, while the test speed was 1.0 mm s^−1^, and the samples were centered and compressed to 75% of deformation. The crosshead speed was set to 5 mm s^−1^ for the first and the second bites, respectively. The data for hardness values were recorded. All tests were performed on at least 15 samples for each treatment.

### 4.5. Respiration Rate

Respiration experiments were carried out at 0, 7, 14, 21, 28, and 45 days of storage time for both uncoated and coated samples. About 50 g of grape tomato samples were placed in a sealed Plexi-glass chamber (18 cm × 12 cm × 27 cm) at room temperature. The chamber was equipped with a CO_2_ sensor (ACR Systems Inc, St-Laurent, Quebec, Canada), connected to a data acquisition system (Smart Reader plus 7). The gas (CO_2_) concentration was measured every 1 min over 2 h, and the respiration rate was obtained from the regression slope of gas concertation versus time. Grape tomato respiration rates were determined as ml CO_2_ kg^−1^ h^−1^ [27].

### 4.6. Microbial Growth

Microbiological growth analysis was carried out on uncoated and coated grape tomato samples and performed on days 0, 7, 14, 21, 28, and 45. Approximately 10 g of each sample was homogenized with 90 mL sterile peptone water (0.1% *w*/*w*). Serial dilutions (10^−1^, 10^−2^, 10^−3^, and 10^−4^) were poured on PCA (plate count agar), a selective media for aerobic mesophilic bacteria. After the plates were incubated for 2 days at 30 °C, colony counts were made. All tests were performed in triplicate.

### 4.7. Statistical Analyses

Statistical analysis of the SPSS software (SPSS, 2023) was used to determine the effect of coating and storage time on the quality and shelf life of grape tomatoes. Differences between treatments were tested for significance by one-way ANOVA. Significant different means (*p* ≤ 0.05) were separated by the Duncan test.

## 5. Results and Discussion

### 5.1. Characterization of Film Properties

#### 5.1.1. Particle Size and Zeta Potential Measurement

Representing particle size, the volume mean diameter (D_3.2_) is the average size of particles within the sample, while the zeta potential measures the stability of colloidal dispersions. The NaCa-CMCH-BO sample showed a higher D_3.2_ value than CaCa-CMCH-BO (295 ± 1.3 vs. 117 ± 0.24, respectively), indicating that the NaCa-CMCH-BO sample had larger particles than the CaCa-CMCH-BO sample. The zeta potential value of the anionic CaCa-CMCH-BO sample was −40.7 ± 1.77, higher than that of the NaCa-CMCH-BO sample at −25.1 ± 0.24, indicating that the calcium caseinate sample offered a more stable colloidal dispersion. The addition of oil also resulted in slightly altering the particle size.

#### 5.1.2. Physicochemical Properties

Thickness: The thickness of composite polymer films must be controlled, as such combinations can lead to thicker films. This will impact other properties (e.g., appearance, barrier, and mechanical) [14]. In practical applications, the optimal thickness of edible films and coatings depends on balancing between mechanical integrity and barrier performance, as well as sensory acceptability. Polymer film thicknesses between 0.05 mm and 0.20 mm are generally considered suitable for edible films and coatings because of their cohesiveness and practicality [14]. However, in general, an optimal film thickness ranges from 0.05 to 0.33 mm, while edible coating is less than 0.025 mm [14]. Heightened moisture content in the films can also increase the film thickness [28]. Polymer films with thicknesses between 0.05 mm and 0.20 mm are considered to be cohesive and thin [14]. Measured film thicknesses ranged between 0.154 ± 0.010 mm and 0.129 ± 0.040 mm (Table 1). Blackseed oil incorporation led to a significant (*p* ≤ 0.05) decrease in edible film thickness compared to BO-free films, but there was no significant difference between the films with or without added oil between NaCa- and CaCa-based films (*p* > 0.05). Shen et al. [29] found that the thickness of a pullulan-gelatin-based edible film decreased when clove (*Syzygium aromaticum* L.) essential oil was added. Similarly, Rocca-Smith et al. [30] found film thickness to be less when lipid was incorporated into wheat-gluten-based edible films.

Water solubility: Film amendment with BO resulted in significant decreases (*p* ≤ 0.05) in the film’s water solubility, moisture content, and WVP values (Table 1). However, the higher solubility of an edible film makes it easy to dissolve in water and reduces its capacity to retain moisture. Conversely, lower solubility indicates that the edible film does not degrade quickly and can be useful for coating food and preventing its damage. Water vapor permeability is one of the most important functions of edible films. The film should minimize the water exchange between the food and the surrounding environment, helping control food spoilage and degradation of product quality. However, using hydrophobic materials like essential oil can help reduce moisture transfer. The incorporation of blackseed oil into the film led to a decrease in WVP, with the lowest value of 3.01 g kPa^−1^ h^−1^ m^−2^ for CaCa-CMCH-BO. Wai et al. [31] reported that blending chitosan with NaCas had the lowest water solubility (27.6% ± 3.9), indicating that composite films would improve water resistance as food. Another research found that incorporating sunflower seed oil (SSO) into the film matrix led to a reduction in the solubility of the films. However, concentrations of 0.5, 1, and 2% (*w*/*w*) of SSO led to a reduction of 18.12, 27.99, and 34.80% in water solubility, respectively [32].

#### 5.1.3. Color and Opacity Index

The color of the edible film can affect how the packaged product looks and influence consumer preference. Table 2 presents the color parameters of the films and their opacity index with and without amendment with blackseed oil. All color parameters were significantly different (*p* ≤ 0.05) when blackseed oil was applied. The blackseed oil effect of the final color of the dried film (more yellow) can be confirmed by the b* value. The b* value of film samples containing essential oil (BO) was 31.1 for CaCa-CMCH-BO and 23.5 for NaCA-CMCH-BO; the values were higher for oil-containing films compared with BO-free samples. The ∆E value ranged from 6.09 to 35.3 and was higher in BO-amended samples than in control BO-free samples. This increase in ∆E was mainly influenced by the variation in the b* value. Similar results were obtained for yellow index results. The YI is affected by both L* and b* values, which were higher in the BO-amended sample. For the prepared films, the L* values were greater in the control (91.2 and 91.8) than for films amended with BO (82.6 and 87.7). Thus, the oil incorporation decreased the edible film’s transparency. Zhang et al. [33] found that using cinnamon bark oil and soybean oil had the same effect as we described when incorporated into an alginate edible film. Likewise, Chen et al. [34] showed the amendment of a chitosan-pullulan edible film with *Artemisia annua* L. essential oil led to an increase in the ∆E value and b* value of treated films.

The opacity index of the edible film influences both the visual and functional properties of packaging materials. The light transmission of the edible films was determined by absorbance measurement, and then the opacity was calculated. Higher opacity indicates that the films had a lower transparency. The opacity index of the samples with BO-amended films was significantly greater (*p* ≤ 0.05) than that of the BO-free films (Table 2). Thus, adding the BO led to a significant increase in the edible film opacity. The opacity indices of the films with BO were 1.22 and 2.36 nm mm^−1^, respectively.

#### 5.1.4. Mechanical Properties

**Figure 1:**
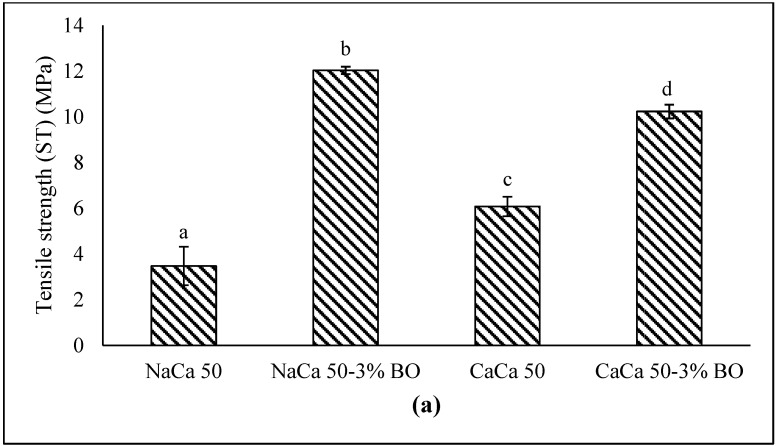

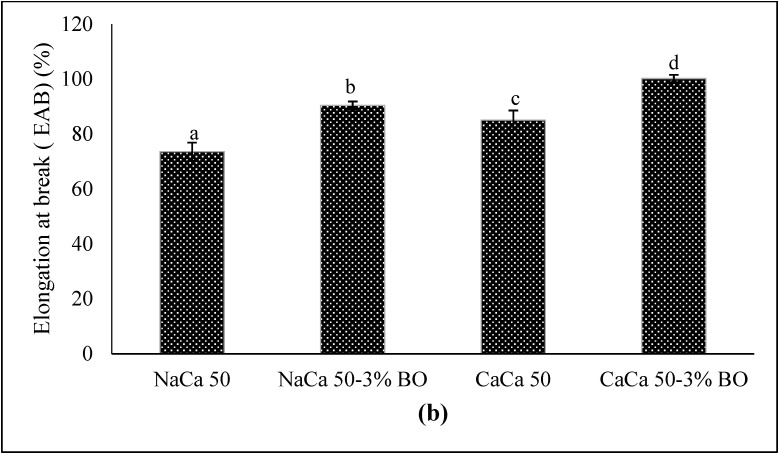
Mechanical properties of caseinate–carboxymethyl chitosan (CAs-CMCH) composite-based edible film incorporated with (3%) blackseed oil or without BO. (**a**) Tensile strength, (**b**) Elongation at break. Vertical bars represent standard error. Bars with the different letter indicate significant differences at (*p* ≤ 0.05).

Understanding materials’ mechanical properties helps to predict their behavior under different forces. Tensile strength (TS) and elongation at break (EAB) are among the most important of such parameters. The TS indicates how strong materials are by quantifying what force can be applied before they break, while EAB measures how flexible the materials are when stretched. Adding blackseed oil into the edible film matrix led to a significant (*p* ≤ 0.05) increase in the mechanical properties measured (Figure 1). The TS (12.5 MPa and 10.2 MPa for NaCa-CMCH-BO and CaCa-CMCH-BO, respectively) and EAB (90.5% and 100% for NaCa-CMCH-BO and CaCa-CMCH-BO, respectively) and values were higher in BO-amended samples compared with BO-free control samples. This increase in TS was acceptable since the standard value of the tensile strength of the film packaging exceeded 3.5 MPa [14].

In contrast to using a specific level of lipid in an edible film composite matrix to positively affect mechanical behavior, essential oils, in proportionally lower amounts, seem to show some incipient ability to reduce the intrinsic stiffness of protein films, thereby enhancing their mechanical properties. However, the type of essential oil significantly affects both TS and EAB. Moreover, the inclusion of an essential oil can increase a film’s flexibility by disrupting polymer chain interactions, causing reduced polymer bonding. This result has been noted with a range of essential oils: lemon (*Limon* (L.), ginger (*Zingiber officinale* Roscoe), peppermint (*Piperita* L.), bergamot (*Citrus bergamia* L.), sweet orange (*Citrus* (L.), and grapefruit. The present study found that BO amendment resulted in both a more elastic and stronger edible film. This concurred with the findings of [34]; the incorporation of cinnamon bark oil and soybean oil into alginate films resulted in tensile strength (TS) values of 16.0 MPa and 16.0 MPa, respectively, while the elongation at break (EAB) values were 36.32% and 46.0%, compared to the control, which exhibited a TS of 6.51 MPa and an EAB of 12.0%. Similarly, other studies [34,35,36] found that by incorporating an essential oil into the film formation solution, the edible film’s mechanical properties were enhanced.

#### 5.1.5. Antioxidant Activities

The incorporation of essential oils into the film matrices has recently garnered significant attention, owing to their natural antioxidant properties. The use of biodegradable, edible films in the food packaging industry serves as an alternative to synthetic plastic bags in offering protection for food products. Plant essential oils are rich in bioactive compounds (e.g., phenolics and flavonoids), which contribute to their antioxidant activity. Adding essential oil to these films can preserve the quality of food by inhibiting lipid oxidation, delaying spoilage, and extending the shelf life of the food. The antioxidant capacity of edible film samples containing blackseed oil (BO) was evaluated with DPPH and ABTS assay. The incorporation of BO into the film samples enhanced both DPPH and ABTS scavenging activity (*p* ≤ 0.05). The antioxidant activity of the NaCa-CMCH-BO film was greater than that of the CaCa-CMCH-BO, while the Ca and Na films showed no difference between (p> 0.05). The antioxidant activity contributed by the CaCa and NaCa films were 20.3%, 16.8% for DPPH, respectively, and 22.4%, 20.03% for ABTS. This could be due to some antioxidant activity of the BO-free edible film composite polymer or the chemical interaction between two polymers. Compared to the control, BO-containing films showed scavenging activities of 55.2% and 40.2% for DPPH and 60.1% and 46.4% for ABTS, respectively. Several studies have investigated the effect of essential oil amendment on edible films, including clove oil [30], galangal oil [36], persicaria minor Hud’s oil [37], garlic, turmeric, and kaffir lime oil [38], black pepper oil [14], Clary sage oil [39], and lemongrass oil [39].

#### 5.1.6. FTIR Analysis

**Figure 2:**
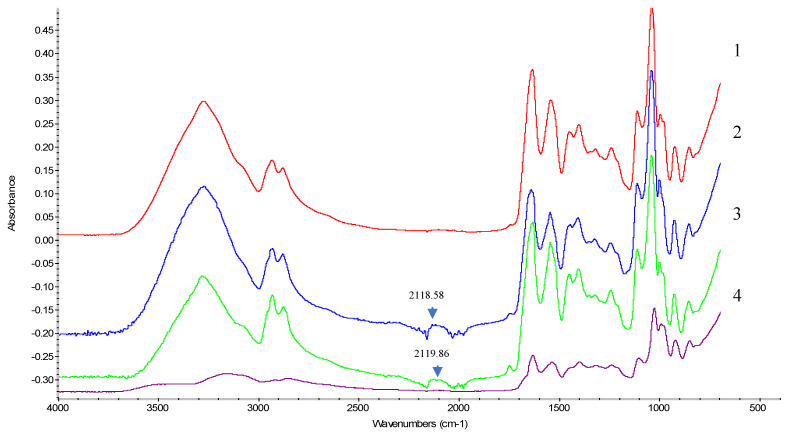
FTIR spectrum of caseinate–carboxymethyl chitosan (CAs-CMCH) based edible film samples in presence or absence of blackseed oil at 3% BO.

**Figure 3:**
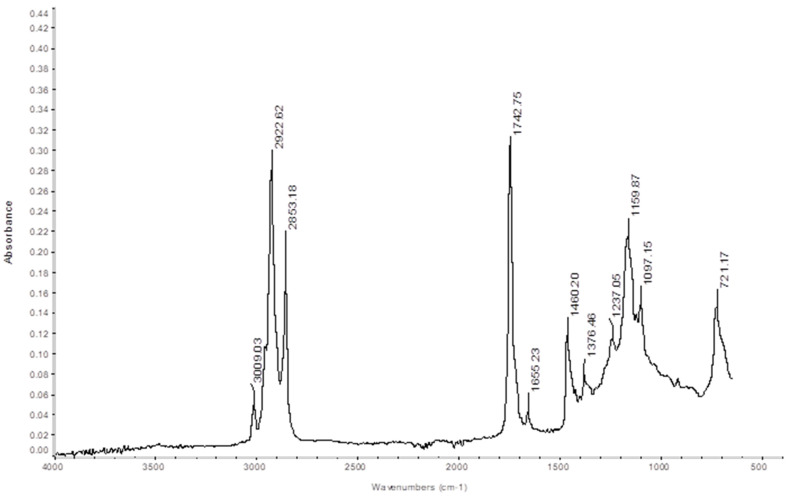
FTIR spectrum of fresh blackseed oil (BO).

FTIR analysis of composite caseinate–carboxymethyl chitosan-based edible film with and without (BO) presented in Figure 2 and Figure 3 highlights the pure blackseed oil (BO) spectrum scanned at 4000–500 cm^−1^. The results of FTIR spectra of pure BO have assigned the presence of various sharp, strong, and weak peaks, with crucial functional groups including C-H, -CH_2_, -CH_3_, C=C, and C-O [11]. Peaks include a strong absorption at 2922.62 cm^−1^ and 2853.18 cm^−1^, attributed to the C–H stretching of an aliphatic group, indicating the existence of methyl and isopropyl substituents. A sharp peak at 1742 cm^−1^ corresponds to the C=O stretching in esters or fatty acids. The peak at 1655 cm^−1^ suggests the presence of C=O stretching of TQ (Thymoquinone) because of the decrease in the resonance frequency effect of the carbonyl group, likely from unsaturated fatty acids. Meanwhile, the bands at 1460 cm^−1^ and 1376 cm^−1^ can be assigned to bending vibrations of CH₂ and CH₃ groups. Moreover, the peaks at region 1237 cm^−1^ (C–O stretching) and 1159 cm^−1^ indicate ester functionalities, and the absorption at 721 cm^−1^corresponds to long-chain aliphatic compounds. The weak absorption peak at 3009 cm^−1^ could correspond to the C-H stretching of the vinyl group, as well as the band at 1097 cm^−1^, owing to a = C-H bending group. All these findings are very similar to those found by Mohammed et al. [11].

The FTIR spectrum of the samples of caseinates with carboxymethyl chitosan blend revealed critical interactions and functional groups after adding the blackseed oil. Each spectrum emphasized a unique peak corresponding to functional groups and their potential interactions. Peaks in the 3000–3500 cm^−1^ region represent O-H and C-H stretching vibrations, with a slight change in shift between NaCa 50 and NaCa 50–3% BO (3270 cm^−1^ vs. 3276 cm^−1^), compared to CaCa 50 and CaCa 50–3% BO samples (3150 cm^−1^ and 3270 cm^−1^). These shifts are indicative of protein backbone and polysaccharide interaction. All samples except the CaCa showed a band at an asymmetric stretch around 2930 cm^−1^, whereas the CaCa showed a symmetric stretch around 2851 cm^−1^. Those showed overlap and intensified, with excitation peaks confirming a successful interaction between the components. New peaks appear at 2118 cm^−1^ and 2119 cm^−1^ for NaCa-CMCH-BO and CaCa-CMCH-BO, respectively, representing the C=C stretch [40].

Peaks at 1600–1700 cm^−1^ (amide I) and 1000–1550 cm^−1^ (amide II) emerge from the proteins (caseinates) structure, while peaks at 1400 cm^−1^ refer to COO^-^ stretching, and peaks at 1100–1200 cm^−1^ to C-O stretching vibrations, both from polysaccharides (CMCH) [41,42]. New peaks and shifts appear in the 600–1500 cm^−1^ region when the oil is added, which suggests that there is a chemical interaction between the three materials (e.g., hydrogen bonding between the hydroxyl groups (Figure 2). In summary, through the intensities and positions of the peaks in the areas corresponding to O-H, C=O, and C-H, the FTIR results confirm the effectiveness of the blending of caseinates and carboxymethyl chitosan and blackseed oil, illustrating their significant interaction.

#### 5.1.7. Scanning Electron Microscopy (SEM)

**Figure 4:**
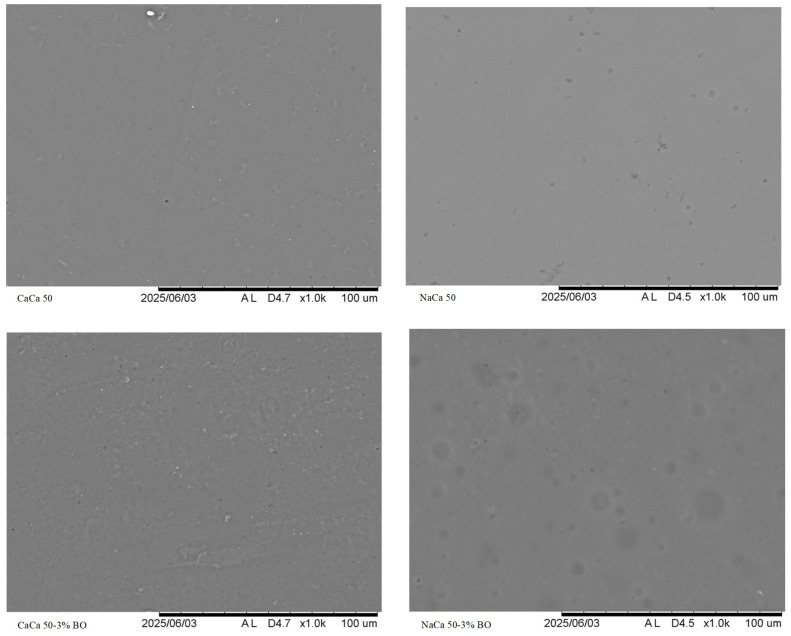
Scanning electron micrographs (SEM) of caseinate–carboxymethyl chitosan (CAs-CMCH) composite-based edible film at ratio (50:50) with and without blackseed oil at 3% BO; surfaces (Magnification: 1000×).

The analysis of surface micrographs of the film samples with and without the addition of BO. The image showed that the surface was relatively smooth, suggesting good film formation with no phase separation. The SEM results of the control BO-free sample showed slight bumps in the smooth surface due to the effect of the caseinate cross-linking, with small particles visible on the film surface. Amendment with BO made the film smoother and without cracks or pores, which may be attributed to adding Tween 80. However, using BO in the film matrix also led to changes in the morphology of the film, which could enhance certain of its properties, as confirmed by the mechanical and barrier results as shown in Figure 4.

## 6. Quality Parameters of Tomatoes with and Without Coating

### 6.1. pH, Total Soluble Solids, Titratable Acidity, and Weight Loss

In the present study, various physical properties of the edible-film-coated tomatoes and the uncoated controls were investigated, including pH, total soluble solids (TSS), and titratable acidity (TA) (Table 3). These parameters were analyzed to understand how they might reflect the metabolic status of grape tomatoes, a key factor in maintaining fresh food quality. In general, for all samples, pH values increased with the storage time. Uncoated samples started with a pH = 4.83 and gradually increased to 5.16 at 45 days. This rise indicates that the control tomato samples were becoming less acidic, which has been attributed to microbial activity or enzymatic breakdown converting organic acids into less acidic compounds. In comparison, the coated tomatoes showed an increase from 4.81 to 5.08 for NaCa-CMCH-BO and 4.81 to 5.02 for CaCa-CMCH-BO, suggesting that the treatment was effective in slowing down the rise in pH. The presence of this coating could be inhibiting microbial growth and enzymatic activity. The CaCa-CMCH-BO coating was the most effective in maintaining the grape tomatoes’ acidity, offering the potential for edible coating.

Coated grape tomatoes exhibited a lesser increase in total soluble solids (TSS) over 45 days of storage compared with uncoated samples. The initial TSS values for all samples were similar (≈4.3%), allowing a direct comparison of the coating’s effects on the TSS (Table 3). However, over time, the uncoated samples showed a consistent increase, reaching 5.30% by the end of the storage period. In contrast, tomatoes coated with NaCa-CMCH-BO and CaCa-CMCH-BO reached TSS levels of 4.89% and 4.91%, respectively, suggesting that the coating treatment could help delay ripening rates by providing a barrier to water loss and potentially preserving cell integrity. The CaCa-CMCH-BO showed the lowest TSS increase, highlighting its potential for extending shelf life and maintaining quality in fresh produce by controlling metabolic changes that lead to ripening.

The main acids in grape tomatoes are ascorbic and citric acid, accounting for most of their titratable acidity [43]. Over 45 days of storage, both treated and untreated samples showed changes in TA. All samples showed similar initial TA levels of 0.695% (Table 3). However, there was a significant difference (*p* ≤ 0.05) between coated and uncoated samples in the tests on the same days, except at 45 days when there was no difference between the uncoated (control) and CaCa-CMCH-BO. During the storage, the TA decreased for all samples, with the uncoated sample (control) grape tomatoes showing a greater decrease in TA, reaching 0.44% at 45 days. The coated samples showed a slightly greater decline, reaching 0.43% for NaCa-CMCH-BO and 0.38% for CaCa-CMCH-BO, demonstrating a more significant preservation of acidity at 45 days.

Other studies have demonstrated that using coating edible films can reduce the number of microorganisms and delay ripening, which contributes to the extension of tomato shelf life. Pobiega et al. [44] reported that using pullulan with propolis extract could improve the physicochemical properties of cherry tomatoes. Moreover, [45] applied carboxymethylcellulose-based composite coating containing cinnamaldehyde and zinc oxide nanoparticles on cherry tomatoes to improve their postharvest quality. They found that the results suggested using the ZnO/CIN/CMC nanocomposite film packaging could improve the cherry tomatoes’ quality by suppressing the physiological and metabolic activities of the fruits during the postharvest storage period.

**Figure 5:**
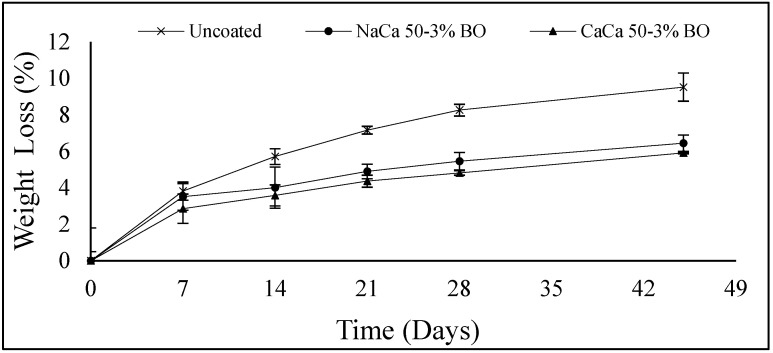
Effect of uncoated and coated treatment on weight loss of grape tomatoes using caseinate–carboxymethyl chitosan (CAs-CMCH) composite-based edible with 3% blackseed oil (BO).

Loss of mass and shrinkage are critical factors influencing shelf life, marketability, and consumer acceptability of fruits and vegetables. These issues are mainly caused by water loss and respiration during storage, leading to an overall reduction in freshness. These changes are directly associated with decreased food quality and increased fungal infection [44]. Shrinkage occurs when the size of vegetables or fruits decreases during storage, which is mainly caused by the evaporation and transpiration of water. As this transfer of moisture occurs through natural pores and respiration, hydrophobic essential oils can improve the coating on the food’s surface, making it less permeable to water. This helps reduce water loss, weight loss, and shrinkage. Acceptable mass loss during storage can vary based on the type of food product and storage conditions; a typical mass loss limit is considered to be 5–10% [46]. Beyond this level, excessive water loss can lead to shrinkage, textural changes, and reduced product marketability. Figure 5 illustrates the mass loss percentage over 45 days for grape tomato samples. In general, there was a significant difference between the coated and uncoated samples on the same testing day (*p* ≤ 0.05). Control samples (uncoated) showed the highest mass loss, starting at 0% and reaching 9.52% by the end of the storage period (45 days). Samples coated with NaCa-CMCH-BO and CaCa-CMCH-BO significantly reduced mass loss by 6.45% and 5.92%, respectively, at the end of storage. This suggests the coating process effectively slowed down the weight loss of the grape tomatoes, more so with the CaCa-CMCH-BO treatment. In another study, the use of a pectin solution to coat tomatoes was found to reduce weight loss to less than 5–6% after 30 days of storage compared with uncoated tomatoes [47]. Similar findings were reported by [13,48], indicating a significant difference in the mass loss between coated and uncoated tomatoes.

### 6.2. Color Parameters

Grape tomatoes are a fruit that continues to ripen after being harvested from the field. Tomatoes quickly become overripe, reducing their quality. The red appearance of the fruit is a key quality attribute, as it is related to freshness, ripeness, and nutritional value. Changes in the color parameters of fruit during storage are considered an important factor, as visual quality affects consumer acceptability. However, fruits are susceptible to color change during storage through natural metabolic processes, enzymatic interactions, and oxidative stress. The L* (lightness), a* (green/red), and b* (blue/yellow) color parameters are used to evaluate tomato color, which can impact their visual appeal, reducing fruit acceptability and marketability. The color change in the tomato samples for both uncoated and coated fruit (Table 4, shows the L* value of control samples to have experienced a significant reduction over 45 days of storage, while those coated with CaCa-CMCH-BO showed a relatively stable value. However, all treatments over time showed a decrease in lightness; however, the NaCa-CMCH-BO remained consistently distinct due to its lower initial L* value. Initial a* values were significantly higher in control compared with coated samples, and over time, all treatments decreased. Similar results were recorded for the b* value after 45 days of storage. The a*/b* values of the control sample were significantly (*p* ≤ 0.05) greater than those of the treated samples, indicating that the coating process led to the tomatoes maintaining their color during the storage time. However, based on USDA recommendations, this color ratio should be between 0.95 and 1.21 [49]. The significant changes in a and b values are related to the behavior of pigment synthesis, and these changes correspond to the massive synthesis of carotenoids [48].

### 6.3. Total Phenolic and Ascorbic Acid

**Figure 6:**
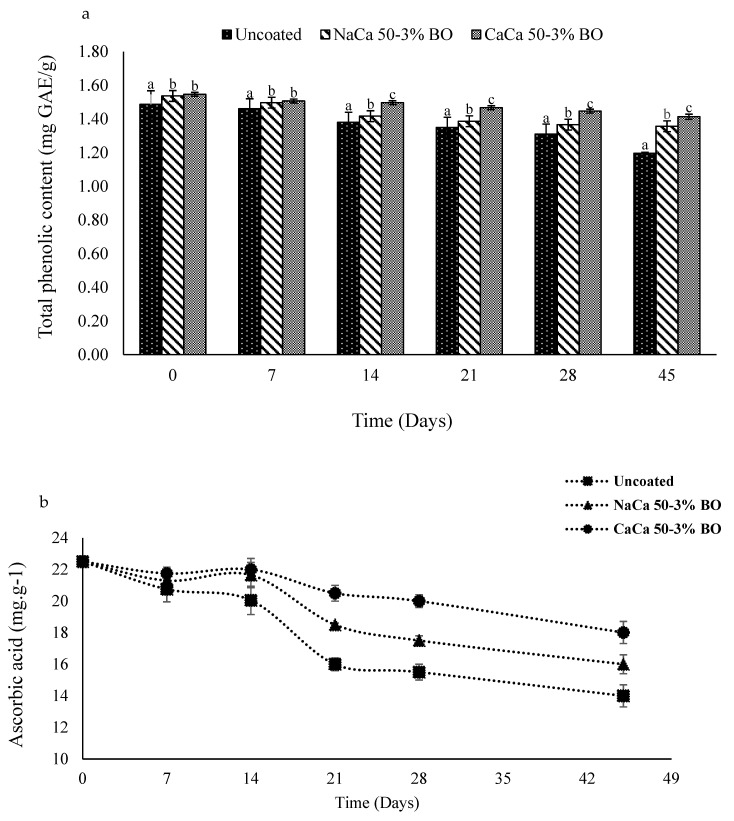
(**a**) Total phenolic content (TPC) and (**b**) Ascorbic acid (AAs) of uncoated and coated grape tomatoes with caseinate–carboxymethyl chitosan (CAs-CMCH) composite-based edible with blackseed oil 3% BO.

Phenolic compounds are characterized by the presence of one or more phenolic groups, which can exist as monophenols, diphenols, triphenols, or polyphenols. They are the most abundant secondary metabolites in plants, assuming a key role in pigmentation, development, and generation of the plant, along with protection from pathogens. The total phenolic content (TPC) in plants varies depending on species, environmental conditions, and temperature. During storage, total phenolic compounds (TPC) generally decline over time [24]. They exhibit diverse chemical and biological properties, such as being antioxidative, anti-cancer, anti-inflammatory, antiobesogenic, anti-allergenic, anti-viral, anti-thrombotic, and antimicrobial, among other properties [50]. Initially, TPC values were slightly higher in coated samples than in uncoated control samples (Figure 6a). This difference was the result of the protective coating shielding the tomatoes from the surrounding environment, thereby slowing enzymatic oxidation and preserving the phenolic compounds, which are naturally prone to degradation over a period of storage. Initially, the uncoated sample contained 1.48 mg GAE g^−1^, a level which declined over time, reaching 1.19 mg GAE g^−1^ by Day 45, a loss of approximately 20%. In contrast, the coated samples showed slower degradation, with TPC reductions of around 12% and 9% over the storage period for NaCa-CMCH-BO and CaCa-CMCH-BO films, respectively. The coating of the samples was observed to reduce the degradation rate of phenolics by creating a barrier against oxygen and moisture, thereby minimizing conditions prone to accelerating phenolic degradation. The CaCa-CMCH-BO coating showed the greatest protective qualities. The ability of the calcium ions to build up strong cross-linkage contributed to its enhanced barrier and mechanical properties, effectively leading to lower oxidative stress. A previous study showed calcium-based coatings to have the ability to delay phenolic oxidation by stabilizing cell walls and inhibiting enzymatic activity [51].

The ascorbic acid content in the fruit increased as the fruit matured, and when the fruit reached full ripeness, the level of ascorbic acid decreased as senescence progressed. The level of AA_s_ increased at the early stages of storage (until Day 14) but began to decline after 21 days (also shown in Figure 6b). This could be due to the grape tomatoes still generating respiratory metabolism [52]. The uncoated samples exhibited faster AA_s_ degradation, with AA_s_ levels dropping to14 mg g^−1^ by the end of storage. In contrast, the coated samples showed significantly lower AA_s_ level degradation, with 16 mg g^−1^ and 18 mg g^−1^ at 45 days of storage for NaCa-CMCH-BO and CaCa-CMCH-BO, respectively. This indicates that the coating process effectively preserved the AA_s_. These results highlight the potential of such coatings in enhancing the shelf life and nutritional quality of food products. Jhanani et al. [13] mentioned that tomatoes coated with pectin (Zucchini) edible coating solution retained more significantly AA_s_ compared to control samples. After 11 days of storage, AA_s_ levels were 21.49 mg g^−1^ and 25.79 mg g^−1^ for 3% and 5% pectin levels, respectively, while uncoated fruit contained 9.68 mg g^−1^ of AA. Another study reported that incorporating crude green algal ethanolic extract (CAEE) into a chitosan-based edible coating helped maintain the level of AA_s_ during storage as compared with an uncoated control [53].

### 6.4. Texture

Texture is a critical factor in determining the quality and consumer acceptability of fresh and processed foods. It plays an important role throughout the food supply chain in deciding when to harvest, evaluating how postharvest handling and processing affect shelf life, and influencing consumer preferences.

**Figure 7:**
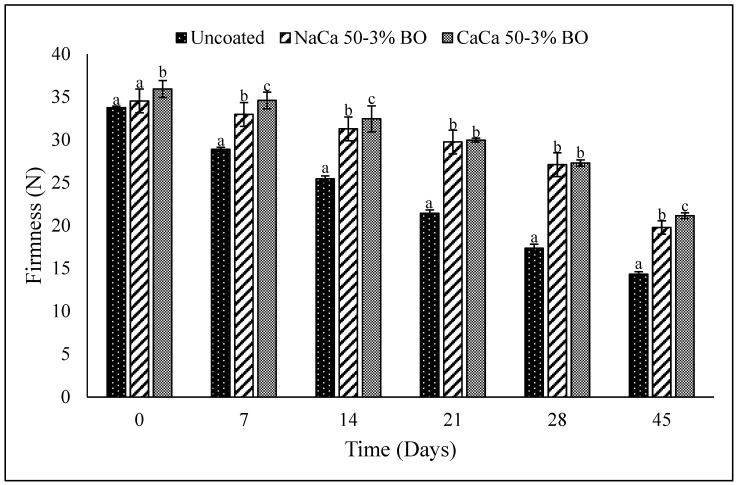
Firmness (N) of uncoated and coated grape tomatoes with caseinate–carboxymethyl chitosan (CAs-CMCH) composite-based edible with blackseed oil 3% BO.

Food texture has been defined as “all the rheological and structural (geometric and surface) attributes of the product perceptible by means of mechanical, tactile, and, where appropriate, visual and auditory receptors” [54]. The firmness was used as an indicator of both the ripeness and freshness of the grape tomatoes. Over a period of storage, the cell structure will degrade, which is produced by gas (carbon dioxide and oxygen) [55]. Textural changes for coated and uncoated (control) tomato fruit samples during storage (Figure 7) show that initially (Day 0), all samples had similar firmness levels, with the coated samples showing a slightly greater firmness than the uncoated controls, though the difference was not statistically significant (*p* > 0.05). As storage progressed, the uncoated tomatoes experienced a rapid decline in firmness, with a final value of 14.33 N by Day 45, indicating that more softening and quality degradation occurred than in the NaCa-CMCH-BO (19.76 N) and CaCa-CMCH-BO (21.14 N) fruit at 45 days. The CaCa-CMCH-BO treatment demonstrated the best performance in maintaining firmness, likely due to the calcium-based coating enhancing structural integrity and reducing moisture loss. These findings concur with previous research demonstrating that tomatoes coated with konjac glucomannan-curdlan edible coating solutions preserved their firmness more effectively than uncoated samples [52]. Similarly, it has been reported tomatoes coated with whey protein isolate/xanthan gum with clove oil retained their firmness better than the uncoated control group [24], aligning with the findings of the present study. Consistent with these results, previous studies have also shown that the plum (*Prunus* sp.) fruit treated with vegetable wax and a shellac-based coating maintained greater firmness compared to an untreated group.

### 6.5. Respiration Rate

**Figure 8:**
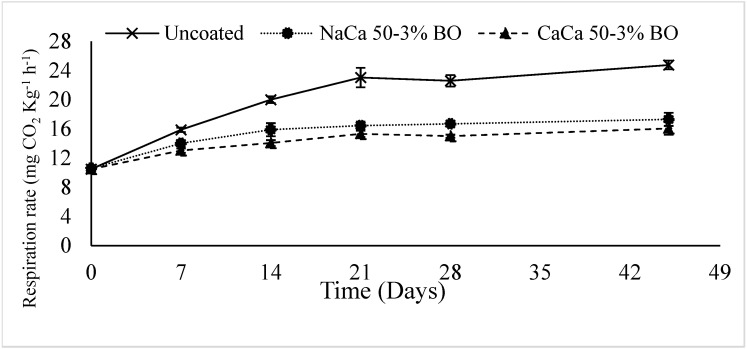
Respiration rate (RR) of uncoated and coated grape tomatoes with caseinate–carboxymethyl chitosan (Cas-CMCH) composite-based edible with blackseed oil 3% BO.

Respiration rate (RR) is one of the main keys to determining food freshness and the ripening stage. Higher RR in fruits and vegetables accelerates metabolic activity and the ripening process, ultimately reducing their shelf life. Increasing RR over the period of storage, as indicated by CO_2_ evolution, was observed in all samples (Figure 8). The uncoated samples exhibited the greatest RR during the storage period, with a stable increase from 10.5 mg CO_2_ kg^−1^ h^−1^ at Day 0 to 24.8 mg CO_2_ kg^−1^ h^−1^ by the end of the storage period (45 days). In contrast, the treated samples showed significantly (*p* ≤ 0.05) lower respiration rates, with both coating treatments stabilizing after Day 21. Between these two coated treatments, CaCa-CMCH-BO maintained the lowest respiration rates. This finding suggested that the incorporation of blackseed oil into edible coating solution significantly decreased the respiration rate, with the CaCa-CMCH-BO treatment providing effective results. Adding the BO to the formulation enhanced its gas barrier properties, likely due to its antioxidant and antimicrobial properties. Furthermore, this characteristic of BO could also help to shield the tomatoes from oxidation and spoilage, effectively slowing down the tomatoes’ respiration rates. Such declines in RR have been reported in various food products: tomatoes [53], sweet cherries (*Prunus avium* L.) [56], and avocado (*Persea americana* Mill.) [57]. 

### 6.6. Microbial Quality

**Figure 9:**
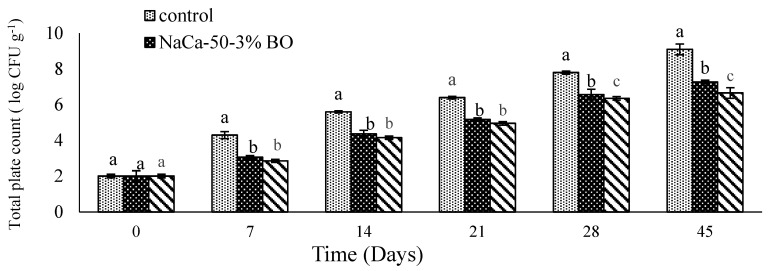
Microbial growth of uncoated and coated grape tomatoes with caseinate–carboxymethyl chitosan (Cas–CMCH) composite-based edible with blackseed oil 3% BO.

The plate count method is the best method to perform microbiological analysis. The effect of blackseed oil and caseinate–carboxymethyl chitosan edible film on microbial growth during the storage of fresh grape tomatoes is illustrated in Figure 9. Initially, all samples showed a similar microbial count of roughly 2 log CFU g^−1^. Over the storage period, the uncoated sample exhibited a steady increase in microbial growth, and it was about 9 log CFU g^−1^ by the end of the storage period (45 days). Compared with the uncoated control, the treated sample had a significantly lower microbial growth at 7.27 log CFU g^−1^ for NaCa-CMCH-BO and 6.6 log CFU g^-1^ for CaCa-CMCH-BO. This could be attributed to the lower permeability of the coating solution, and the antimicrobial activity observed in coated fruit could be due to the presence of blackseed oil. Jin et al. [49] demonstrated that the use of organic acids and essential oils in chitosan-based edible coatings effectively reduced populations of nalidixic acid-resistant *Salmonella*, *Listeria monocytogenes*, and native microorganisms on the surface of whole grape tomatoes.

### 6.7. Visual Appearance

**Figure 10:**
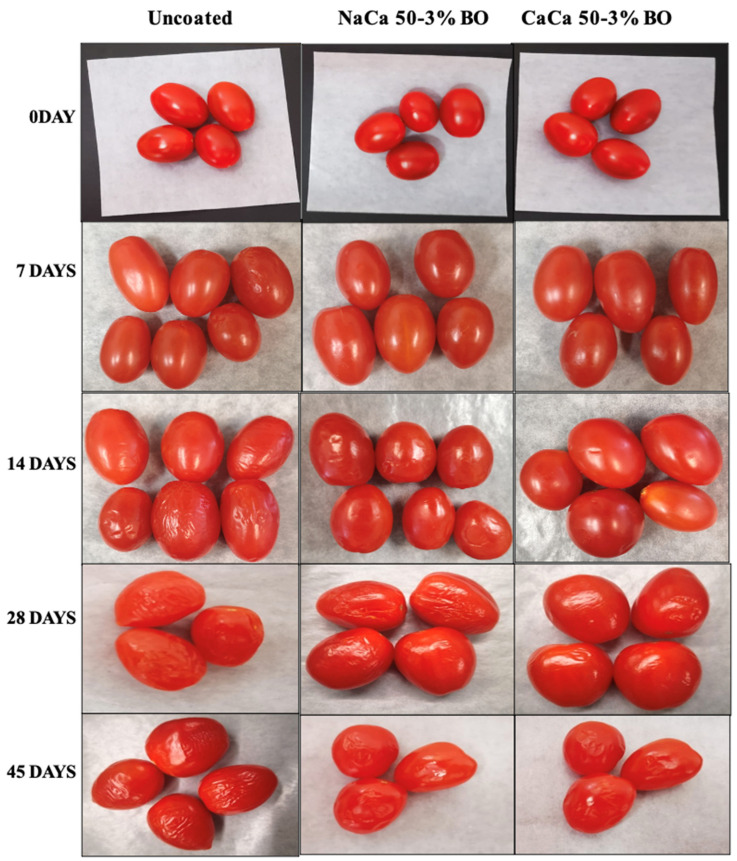
Visual appearance of uncoated and coated grape tomatoes with caseinate–carboxymethyl chitosan (CAs-CMCH) composite-based edible with blackseed oil 3% BO.

Figure 10 shows the visual appearance of edible coated and uncoated grape tomatoes stored at 4 °C for 45 days. Early in storage, the skin of tomatoes was mold-free and smooth. At 7 days, the uncoated (control) samples showed a slight sign of dehydration and wrinkling, while the coated samples maintained a smoother surface, meaning better moisture retention and less shrinkage. After 21 days of storage, the coated samples exhibited moderate signs of dehydration, with CaCa-CMCH-BO coated tomatoes remaining in the best condition, with minimal wrinkling, demonstrating that the coating process effectively preserved the tomatoes’ appearance over time. On the same day, uncoated tomatoes displayed severe wrinkling and shriveling due to substantial water loss and deterioration.

## 7. Conclusions

Biopolymer-based edible films and coatings constitute an eco-friendly, sustainable, low-cost, nontoxic, biocompatible, edible packaging solution. This technique has gained significant attention from researchers and industries due to the growing global demand for alternatives to conventional plastic packaging and the need to improve the postharvest quality of perishable foods. The postharvest shelf life of fruits and vegetables can be prolonged by edible coatings, which create a semi-permeable barrier between the food product and the surrounding atmosphere, thereby helping in controlling the exchange of gases and water vapor, effectively extending their shelf life. In this study, the CaCa-CMCH-BO edible film and coating were successfully prepared using casting and dipping methods. Using BO in the film-forming solution showed an apparent improvement in compatibility, hydrophobicity, and water barrier properties. The film exhibited a tensile strength of 12.5 MPa and 10.2 MP, elongation at break of 90.5% and 100%, and WVP of 4.33 and 3.0 g kPa^−1^ h^−1^ m^−2^ for NaCa-CMCH-BO and CaCa-CMCH-BO respectively. Moreover, the CaCa-CMCH-BO coating maintained the quality of grape tomatoes significantly better than the absence of a coating. Weight loss was reduced by 32.24% and 37.80%, and firmness was retained at 137.9% and 147.5% at 45 days for NaCa-CMCH-BO and CaCa-CMCH-BO, respectively. Therefore, CAs-CMCH edible coatings can be effectively used to extend the shelf life of grape tomatoes and have the potential for fruit and vegetable preservation in the future.

## Figures and Tables

**Table 1:** Nomenclature and physicochemical properties of edible films.

Sample	Thickness (mm)	Water Solubility (%)	Moisture Content (%)	WVP (g kPa^−1^ h^−1^ m^−2^)
NaCa-CMCH	0.154 ± 0.01 ^a^	37.01 ± 0.70 ^a^	21.30 ± 0.11 ^a^	6.62 ± 0.60 ^a^
NaCa-CMCH-BO	0.130 ± 0.02 ^b^	22.20 ± 0.11 ^b^	12.65 ± 0.02 ^b^	4.33 ± 0.08 ^b^
CaCa-CMCH	0.151 ± 0.10 ^a^	31.08 ± 0.32 ^c^	16.44 ± 0.06 ^c^	6.01 ± 0.40 ^c^
CaCa-CMCH-BO	0.129 ± 0.04 ^b^	25.09 ± 0.29 ^d^	10.33 ± 0.08 ^d^	3.01 ± 0.04 ^d^

All values are expressed as mean ± SD. sample means with different superscripted letters in the same column are significant different (*p* ≤ 0.05).

**Table 2:** L*, a*, and b* color parameters, total color difference, yellowness index, and opacity index of edible films.

Sample	L*	a*	b*	∆E	YI	Opacity Index
**NaCa-CMCH**	91.2 ± 0.06 ^a^	−1.14 ± 0.41 ^a^	4.75 ± 0.58 ^a^	7.45 ± 0.72 ^a^	6.87 ± 0.83 ^a^	0.63 ± 0.02 ^a^
**NaCa-CMCH-BO**	82.6 ± 0.67 ^b^	2.08 ± 0.15 ^b^	31.1 ± 0.06 ^b^	35.3 ± 0.31 ^b^	44.9 ± 0.01 ^b^	1.22 ± 0.13 ^b^
**CaCa-CMCH**	91.8 ± 0.20 ^a^	−0.95 ± 0.05 ^c^	3.44 ± 0.09 ^c^	6.09 ± 0.10 ^c^	4.98 ± 0.13 ^c^	0.37 ± 0.03 ^c^
**CaCa-CMCH-BO**	87.7 ± 1.87 ^c^	0.49 ± 1.01 ^d^	23.5 ± 1.55 ^d^	26.5. ± 0.43 ^d^	33.9 ± 1.03 ^d^	2.36 ± 0.09 ^d^

All values are expressed as mean ± SD. sample means with different superscript letters in the same column are significant different (*p* ≤ 0.05).

**Table 3:** pH, Total soluble solids (TSS), and titratable acidity (TA) of uncoated and coated grape tomatoes with caseinate–carboxymethyl chitosan (CAs-CMCH) composite-based edible with blackseed oil 3% BO.

Treatment		Storage Time (days)					
		0	7	14	21	28	45
pH	Control	4.82 ± 0.01 ^Aa^	4.90 ± 0.01 ^Bb^	4.93 ± 0.01 ^Cb^	5.00 ± 0.02 ^Dd^	5.07 ± 0.04 ^Eb^	5.16 ± 0.01 ^Fc^
	NaCa-CMCH-BO	4.81 ± 0.01 ^Aa^	4.82 ± 0.04 ^Aa^	4.91 ± 0.01 ^Bb^	4.94 ± 0.02 ^Cb^	5.00 ± 0.01 ^Da^	5.07 ± 0.01 ^Eb^
	CaCa-CMCH-BO	4.81 ± 0.01 ^Ab^	4.83 ± 0.01 ^Ca^	4.85 ± 0.01 ^Ca^	4.60 ± 0.01 ^Aa^	4.99 ± 0.01 ^Da^	5.01 ± 0.02 ^Da^
TSS	Control	4.42 ± 0.03 ^Ac^	4.55 ± 0.02 ^Bb^	4.78 ± 0.02 ^Cc^	4.86 ± 0.05 ^Db^	5.11 ± 0.02 ^Eb^	5.29 ± 0.03 ^Fc^
	NaCa-CMCH-BO	4.29 ± 0.01 ^Ab^	4.36 ± 0.02 ^Ba^	4.49 ± 0.01 ^Cb^	4.52 ± 0.02 ^Da^	5.67 ± 0.01 ^Fc^	4.88 ± 0.01 ^Ea^
	CaCa-CMCH-BO	4.22 ± 0.01 ^Aa^	4.35 ± 0.01 ^Ba^	4.47 ± 0.04 ^Ca^	4.52 ± 0.02 ^Da^	4.65 ± 0.01 ^Ea^	4.90 ± 0.02 ^Fb^
TA	Control	0.65 ± 0.05 ^Da^	0.52 ± 0.01 ^Cc^	0.51 ± 0.01 ^Cc^	0.48 ± 0.01 ^Bb^	0.47 ± 0.01 ^Bb^	0.44 ± 0.01 ^Ac^
	NaCa-CMCH-BO	0.65 ± 0.04 ^Da^	0.51 ± 0.01 ^C^b	0.50 ± 0.01 ^Cb^	0.47 ± 0.01 ^Ba^	0.46 ± 0.02 ^Bb^	0.43 ± 0.01 ^Ab^
	CaCa-CMCH-BO	0.65 ± 0.03 ^Da^	0.49 ± 0.01 ^Ca^	0.48 ± 0.01 ^Ca^	0.46 ± 0.01 ^Ca^	0.44 ± 0.02 ^Ba^	0.38 ± 0.01 ^Aa^

All values are expressed as mean ± SD. Different lowercase superscript letters within the same column indicate significant differences between treatments on the same day (*p* ≤ 0.05), while different uppercase superscript letters within the same row indicate significant differences over storage time for the same treatment.

**Table 4:** Effect of edible coating on color parameters (L, a, b) and a/b Ratio of Grape Tomatoes.

Treatments		Days of Storage					
L*		**0**	**7**	**14**	**21**	**28**	**45**
	Control	33.41 ± 0.90 ^a^	32.85 ± 0.02 ^a^	28.06 ± 0.08 ^a^	27.96 ± 0.41 ^a^	25.77 ± 0.33 ^a^	23.93 ± 2.01 ^a^
	NaCa-CMCH-BO	28.11 ± 0.04 ^b^	27.09 ± 0.07 ^b^	26.88 ± 0.8 ^b^	26.19 ± 0.08 ^b^	26.01 ± 0.45 ^a^	25.08 ± 0.21 ^b^
	CaCa-CMCH-BO	32.01 ± 0.02 ^a^	31.50 ± 0.01 ^c^	29.69 ± 0.03 ^c^	31.55 ± 0.52 ^c^	31.02 ± 0.88 ^b^	30.21 ± 0.07 ^c^
a*		**0**	**7**	**14**	**21**	**28**	**45**
	Control	22.30 ± 0.03 ^a^	21.35 ± 0.04 ^a^	20.16 ± 0.7 ^a^	20.09 ± 0.01 ^a^	19.55 ± 0.9 ^a^	18.34 ± 0.16 ^a^
	NaCa-CMCH-BO	18.32 ± 0.01 ^b^	16.15 ± 0.11 ^b^	15.09 ± 0.04 ^b^	18.01 ± 0.21 ^b^	18.88 ± 1.03 ^a^	18.06 ± 0.11 ^a^
	CaCa-CMCH-BO	20.09 ± 0.01 ^a^	20.35 ± 0.06 ^a^	19.36 ± 0.03 ^c^	20.89 ± 0.07 ^c^	21.01 ± 0.70 ^b^	20.99 ± 0.53 ^b^
b*		**0**	**7**	**14**	**21**	**28**	**45**
	Control	19.96 ± 0.06 ^a^	18.16 ± 0.09 ^a^	16.97 ± 0.27 ^a^	15.88 ± 0.07 ^a^	15.33 ± 0.85 ^a^	13.09 ± 0.33 ^a^
	NaCa-CMCH-BO	15.60 ± 0.02 ^a^	13.60 ± 0.02 ^b^	14.22 ± 0.06 ^b^	15.78 ± 0.30 ^a^	15.05 ± 0.11 ^a^	13.83 ± 0.69 ^a^
	CaCa-CMCH-BO	17.18 ± 0.01 ^c^	17.56 ± 0.03 ^a^	16.62 ± 0.06 ^a^	17.59 ± 0.03 ^b^	16.87 ± 0.07 ^b^	16.55 ± 0.08 ^b^
a*/b*		**0**	**7**	**14**	**21**	**28**	**45**
	Control	1.17 ± 0.04 ^a^	1.18 ± 0.06 ^a^	1.19 ± 0.49 ^a^	1.26 ± 0.04 ^a^	1.27 ± 0.87 ^a^	1.40 ± 0.25 ^a^
	NaCa-CMCH-BO	1.17 ± 0.01 ^a^	1.19 ± 0.05 ^a^	1.05 ± 0.05 ^b^	1.14 ± 0.26 ^b^	1.25 ± 0.56 ^b^	1.30 ± 0.40 ^b^
	CaCa-CMCH-BO	1.16 ± 0.01 ^a^	1.16 ± 0.05 ^b^	1.16 ± 0.04 ^c^	1.18 ± 0.04 ^c^	1.24 ± 0.28 ^b^	1.27 ± 0.30 ^c^

All values are expressed as mean ± SD. Different lowercase superscript letters within the same column indicate significant differences between treatments on the same day (*p* ≤ 0.05).

## Data Availability

Data will be made available up on request.
